# Optimization of the EUCAST reference broth microdilution method for echinocandin susceptibility testing of *Aspergillus fumigatus*

**DOI:** 10.1093/jac/dkad306

**Published:** 2023-10-09

**Authors:** Maria Siopi, Panagiota-Christina Georgiou, Spyros Pournaras, Joseph Meletiadis

**Affiliations:** Clinical Microbiology Laboratory, “Attikon” University General Hospital, Medical School, National and Kapodistrian University of Athens, Athens, Greece; Clinical Microbiology Laboratory, “Attikon” University General Hospital, Medical School, National and Kapodistrian University of Athens, Athens, Greece; Clinical Microbiology Laboratory, “Attikon” University General Hospital, Medical School, National and Kapodistrian University of Athens, Athens, Greece; Clinical Microbiology Laboratory, “Attikon” University General Hospital, Medical School, National and Kapodistrian University of Athens, Athens, Greece

## Abstract

**Background:**

Because of the high inoculum (10^5^ cfu/mL) used in the EUCAST susceptibility testing of *Aspergillus* spp., determination of the minimal effective concentration (MEC) of echinocandins is challenging as the morphological differences are subtle.

**Methods:**

The MECs of 10 WT and 4 non-WT *Aspergillus fumigatus* isolates were determined with the EUCAST E.Def 9.4. Plates were inoculated with increasing inocula (10^2^–10^5^ cfu/mL) and after 24 and 48 h of incubation, MECs were determined macroscopically (magnifying mirror) and microscopically (inverted microscope) by two observers, spectrophotometrically (OD at 405 nm) and colorimetrically (absorbance at 450/630 nm after 2 h incubation with 400 mg/L XTT/6.25 μM menadione). The interobserver (between observers)/intermethod (compared with the microscopic method) essential agreement (EA, ±1 2-fold dilution) and categorical agreement (CA) were determined for each inoculum.

**Results:**

Echinocandin-induced microscopic hyphal alterations or macroscopic changes in turbidity were subtle with a 10^5^ cfu/mL inoculum compared with the lower inocula of 10^3^ and 10^2^ cfu/mL, where more distinct changes in turbidity and formation of characteristic rosettes were obvious at the MEC after 48 h. A 10^5^ cfu/mL inoculum resulted in wider MEC distributions (3–6 dilutions) and lower interobserver EA (69%), macroscopic–microscopic EA (26%) and CA (71%) compared with a 10^3^ cfu/mL inoculum (2–3 dilutions, 100%, 100% and 100%, respectively). Spectrophotometric readings using a 10^3^ cfu/mL inoculum showed good EA (57–93%) and excellent CA (86%–100%), while the XTT assay demonstrated excellent EA (93%) and CA (100%).

**Conclusions:**

A 48 h incubation using a 10^3^ cfu/mL inoculum improved echinocandin MEC determination for *A. fumigatus* with the EUCAST method, while the colorimetric assay could allow automation.

## Introduction

Echinocandins are currently used as salvage or combination therapy in patients with invasive aspergillosis,^1,2^ and their use may be increased^3^ in light of the spread of azole resistance in *Aspergillus fumigatus*.^4^ Although they demonstrate a uniform potent activity against *A. fumigatus,* clinical isolates exhibiting reduced susceptibility to echinocandins^5,6^ and breakthrough infections^7–10^ have been reported, underlining the importance of reliable echinocandin susceptibility testing of *A. fumigatus*. Echinocandins are fungistatic against filamentous fungi since their activity is restricted to sites where the fungal cell wall is actively growing, namely hyphal tips and branching junctional cells. Thus, growth of filamentous fungi is not completely inhibited at clinically relevant concentrations. Hence, echinocandin activity is assessed *in vitro* not based on the MIC, but on the minimal effective concentration (MEC), defined as the lowest drug concentration at which morphological alterations (aberrant, short hyphal segments) are observed. MEC determination is quite challenging with the EUCAST E.Def. 9.4 protocol, mainly because of the high inoculum (10^5^ cfu/mL) used,^11^ hindering the microscopic/macroscopic and spectrophotometric detection of morphological alterations occurring after exposure to echinocandins. Indeed, a recent multicentre study conducted in laboratories with mycology expertise revealed that the standard microscopic EUCAST MEC determination was associated with considerable intercentre variation, while the macroscopic MEC reading yielded poor intercentre essential agreement (EA) as well as low categorical agreement (CA) in distinguishing WT from non-WT isolates.^12^

An easily determined, objective and quantifiable *in vitro* endpoint is essential for accurate and reproducible antifungal susceptibility testing (AFST). Although several parameters for testing susceptibility of *Aspergillus* spp. to echinocandins following the CLSI guidelines have been investigated,^13^ no such data on the EUCAST methodology are yet available. Inoculum size, incubation time and reading mode are important parameters for the performance of AFST methods. In addition, polysorbate (Tween) 20, a non-ionic surfactant, has been recently found to affect the MIC determination of a new echinocandin, rezafungin, especially when the MIC values are very low because of the drug’s non-specific binding to plastics.^14^ As Tween 20 is used for inoculum preparations for mould AFST and its final concentration inside the wells may vary depending on whether a light or heavy initial inoculum has been prepared,^11^ Tween 20 may affect echinocandin AFST of *Aspergillus* spp., particularly for anidulafungin and micafungin, for which MECs are very low. This study was undertaken to identify the optimal conditions (inoculum size, time of incubation and Tween 20 concentration) for facilitating EUCAST microscopic/macroscopic MEC determination and to explore the use of spectrophotometric and colorimetric assays as alternative methods enabling automation.

## Materials and methods

### Isolates

A total of 10 WT molecularly identified *A. fumigatus* clinical isolates and 4 non-WT *A. fumigatus* isolates kindly provided by D. Perlin (DPL) possessing elevated MEC values, with (DPL1035-homo^15^) or without (DPL55985, DPL32458,^16^ DPLRG101^16^) known *FKS* alterations, were tested. The isolates were stored in normal sterile saline with 10% glycerol at −70°C until use. As neither epidemiological cut-off (ECOFF) values nor susceptibility breakpoints have been determined for echinocandins and *Aspergillus* spp., isolates cannot be classified as WT/non-WT and susceptible/resistant. However, for simplicity, WT isolates were considered common isolates with MECs ≤ 0.125 mg/L for anidulafungin, ≤0.06 mg/L for micafungin and ≤1 mg/L for caspofungin and non-WT isolates with higher MECs.

### Antifungal drugs, chemical reagents and medium

Laboratory-grade standard powders of anidulafungin (AFG; Pfizer, Inc., Groton, CT, USA), caspofungin acetate (Merck & Co., Inc., Whitehouse, NJ, USA) and micafungin (Astellas Pharma, Inc., Tokyo, Japan) were dissolved in sterile DMSO (Chem-Lab NV, Zedelgem, Belgium) and stock solutions of 10 mg/mL were stored at −70°C. XTT sodium salt (AppliChem, Darmstadt, Germany) was dissolved in sterile water before use. Menadione (Sigma–Aldrich, Steinheim, Germany) was dissolved in absolute ethanol (VWR Chemicals, Fontenay-sous-Bois, France) and stock solutions of 58 × 10^−3^ M were stored at −70°C. The medium used throughout was RPMI 1640 medium (with L-glutamine, without bicarbonate) (AppliChem, Darmstadt, Germany) buffered to pH 7.0 with 0.165 M MOPS (AppliChem, Darmstadt, Germany) and supplemented to a final concentration of 2% glucose (AppliChem, Darmstadt, Germany). Tween 20 (AppliChem, Darmstadt, Germany) at 0.1% concentration was added to facilitate the preparation of initial conidial suspensions before adjusting to the desired inoculum in water, whereas in experiments where the impact of Tween 20 was studied, 0.1%, 0.01% and 0.001% were used for working and final inoculum.

### Broth microdilution (BMD) susceptibility testing

The reference BMD procedure was carried out according to the EUCAST recommendations. Briefly, 2-fold serial drug concentrations ranging from 0.008 to 8 mg/L of all three echinocandins were used. Isolates with low off-scale MECs of anidulafungin and micafungin were retested with lower concentrations ranging from 0.0005 to 0.03 mg/L. Each isolate was revived by subculturing it twice on Sabouraud dextrose agar plates with gentamicin and chloramphenicol (bioMérieux) at 30°C for 5–7 days and conidial suspensions were prepared in sterile water with 0.1% Tween 20. Conidia were then counted in a haemocytometer and diluted in sterile water in order to obtain 2× the final inoculum of 10^2^, 10^3^, 10^4^ and 10^5^ cfu/mL. Plates were inoculated with the increasing inocula and were incubated at 37°C for up to 48 h. The inoculum sizes of all strains were affirmed each time by using quantitative colony counts. *Candida krusei* ATCC 6258 and *Candida parapsilosis* ATCC 22019 were used as quality control strains.

### Microscopic method

The MEC was defined as the lowest echinocandin concentration at which only short, stubby and highly branched hyphal clusters (rosettes) were observed compared with the healthy hyphae in the growth control (GC) well after 24 and 48 h of incubation using an inverted microscope. A second endpoint was defined as the lowest drug concentration with a mixed phenotype of the presence of rosettes and healthy hyphae (mMEC).

### Macroscopic method

The fungal growth in each well was determined by inspection of the plate from the bottom with the aid of a magnifying mirror after 24 and 48 h of incubation. The visual MEC was defined as the lowest drug concentration at which small, rounded, compact hyphal forms were observed compared with the hyphal growth seen in the GC well.

### Spectrophotometric method

The OD of each well was measured spectrophotometrically at 405 nm at a single point (centre of the well).^12^ The % fungal growth was calculated for each well as (OD_drug well_ − OD_background drug well_)/(OD_GC well_ − OD_background GC well_) × 100%. The spectrophotometric MEC was determined as the lowest echinocandin concentration corresponding to 50% percentage of growth inhibition (100%−%growth) compared with the GC.

### Colorimetric method

A recently described XTT method was evaluated.^12^ The XTT cell viability assay is a colorimetric assay that uses the tetrazolium dye XTT in order to detect the fungal metabolic activities and quantify drug-induced cell-mediated damage to fungi via the cellular redox potential in live cells. The absorbance (ABS) at 450/630 nm was measured after 2 h of incubation with 50 μL of 5× XTT/menadione solution (final concentrations 400 mg/L and 6.25 μM) in each well. The % metabolic activity assessed by %XTT conversion was calculated for each well as (ABS_drug well_ − ABS_background drug well_)/(ABS_GC well_ − ABS_background GC well_) × 100%. The colorimetric MEC was determined as the lowest drug concentration corresponding to 50% of inhibition of metabolic activity (100%−% metabolic activity) compared with the GC.

### Effect of Tween 20

Microscopic/macroscopic MECs, as well as spectrophotometric and colorimetric MICs, were determined in the presence of a final concentration of 0.05%, 0.005% and 0.0005% Tween 20 inside the wells for the quality control strains *A. fumigatus* ATCC 204305 and *Aspergillus flavus* ATCC 204304. The standard 0.1% Tween 20 solution was 10-fold serially diluted to obtain 0.01% and 0.001% concentrations in sterile distilled water, which were used to prepare the working and the 2× final inoculum, taking great care to ensure accurate volume transfer owing to the high viscosity of the surfactant, and the working solutions were autoclaved.^14^

### Analysis

MECs were evaluated microscopically and macroscopically by two blinded observers (one experienced and one trainee), and interobserver EA (±1 2-fold dilution) was estimated. The level of intermethod EA (±1 2-fold dilution) was assessed by comparing the microscopic MECs as determined by the experienced observer and the MECs of each method (macroscopic, spectrophotometric and colorimetric). Paired *t*-tests were performed to estimate the significance of EA differences of each evaluated method for all echinocandins (*P* < 0.05 was considered statistically significant). CA in distinguishing non-WT from WT isolates was calculated for each method. Differences between MECs in the presence of different concentrations of Tween 20 were determined.

## Results

### Microscopic method

**Figure 1.**
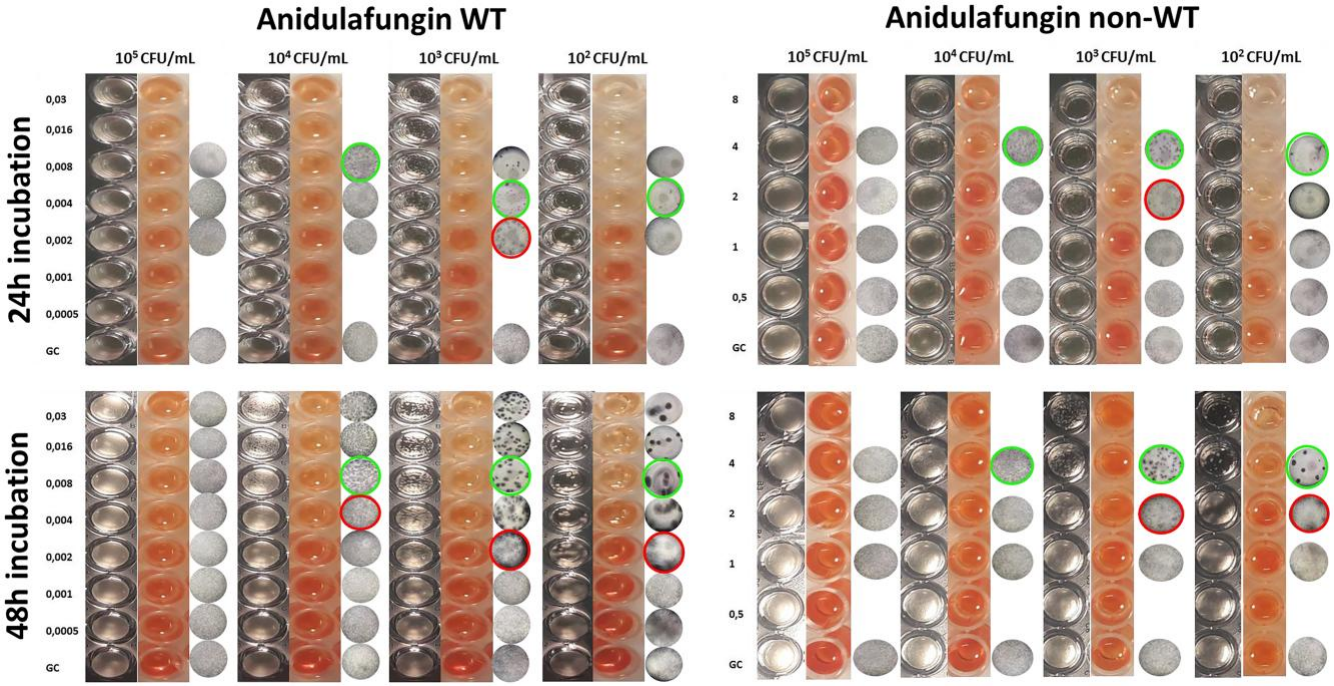
Macroscopic (first column), XTT (second column) and microscopic (third column) evaluation of growth of a WT *A. fumigatus* (left) and a non-WT *A. fumigatus* (right) exposed to 2-fold seral dilutions of anidulafungin (0.0005–8 mg/L) using different inoculum sizes and time of incubation. Green and red circles represent microscopic MEC (mycelial rosettes appear as black dots) and mMEC (mycelial rosettes and healthy hyphae), respectively. This figure appears in colour in the online version of *JAC* and in black and white in the print version of *JAC*.

Insufficient growth was observed for 2/4 non-WT strains using a 10^3^ and 10^2^ cfu/mL inoculum, as well as 3/10 WT strains using a 10^2^ cfu/mL inoculum after 24 h. All isolates grew sufficiently after 48 h and the microscopic MECs were more clearly determined with MECs being ±1 log_2_ dilution from the 24 h MECs. MEC determination was particularly challenging using the standard 10^5^ cfu/mL inoculum since the microscopic differences were subtle (Figure 1, third column). In fact, no clear echinocandin-induced morphological hyphal alterations could be defined microscopically. In absence of typical rosettes, MEC values were reported as the lowest drug concentration where differences in the hyphal density were observed on intense light. The formation of characteristic rosettes was obvious with a 10^4^ cfu/mL inoculum after 48 h of incubation, but differences were more pronounced with either a 10^3^ or a 10^2^ cfu/mL inoculum, where WT isolates could be easily distinguished from non-WT ones. Of note, the second endpoint, mMEC, could be easily identified using the 10^3^ and 10^2^ cfu/mL inocula (Figure 1, third column). The median (range) differences between mMEC and the corresponding MECs were 2 (1–2) and 2 (1–4) dilutions lower for anidulafungin and micafungin, respectively, and 1 (1–2) dilution for caspofungin (ANOVA *P* < 0.05). The average (among the three drugs) interobserver agreement for 48 h MECs was 69%, 82%, 100% and 100% with the 10^5^, 10^4^, 10^3^ and 10^2^ cfu/mL inoculum, respectively.

A 10^5^ cfu/mL inoculum after 48 h of incubation resulted in wider MEC distributions (3–6 2-fold dilutions) compared with a 10^3^ cfu/mL inoculum (2–3 2-fold dilutions) for the WT isolates. (Table 1). The non-WT isolates DPL1035-homo, DPL55985 and DPL32458 had high MECs (≥2 mg/L) for all three echinocandins, except for the spontaneous mutant DPLRG101, which demonstrated a non-WT phenotype only to caspofungin (anidulafungin, micafungin and caspofungin MECs 0.008, 0.008 and 4 mg/L, respectively, for all inoculum sizes tested) (Table 1).

**Table 1.** Comparison of macroscopic and microscopic MECs for WT and non-WT *A. fumigatus* isolates after 48 h of incubation

*A. fumigatus* isolates (*n*)	Drug	Microscopic MEC								% Macroscopic–microscopic agreement (±1 log_2_ dilution)			
		Median (range) MEC (mg/L)				Number of 2-fold dilutions of MEC ranges; % MEC equal to the median MEC							
		10^5^ cfu/mL	10^4^ cfu/mL	10^3^ cfu/mL	10^2^ cfu/mL	10^5^ cfu/mL	10^4^ cfu/mL	10^3^ cfu/mL	10^2^ cfu/mL	10^5^ cfu/mL	10^4^ cfu/mL	10^3^ cfu/mL	10^2^ cfu/mL
WT (10^a^)	AFG	0.016 (0.004–0.125)	0.008 (0.004–0.016)	0.004 (0.004–0.008)	0.004 (0.004–0.008)	6; 50	3; 80	2; 70	2; 70	27	100	100	100
	CAS	0.5 (0.125–0.5)	1 (0.5–1)	0.5 (0.5–1)	0.5 (0.25–1)	3; 50	2; 60	2; 60	3; 50	18	100	100	100
	MFG	0.016 (0.008–0.06)	0.016 (0.004–0.016)	0.008 (0.004–0.016)	0.008 (0.004–0.016)	4; 30	3; 60	3; 70	3; 70	36	91	100	100
non-WT (4^b^)	AFG	2 (2–2)	2 (2–4)	4 (4–4)	4 (4–4)	1; 100	2; 67	1; 100	1; 100	0	100	100	100
	CAS	8 (4–8)	8 (4–8)	8 (4–8)	8 (4–8)	2; 75	2; 75	2; 75	2; 75	0	100	100	100
	MFG	2 (2–2)	4 (2–4)	4 (2–4)	4 (2–4)	1; 100	2; 67	2; 67	2; 67	67	100	100	100
Total	AFG									21	100	100	100
(14)	CAS									140	100	100	100
	MFG									43	93	100	100

AFG, anidulafungin; CAS, caspofungin; MFG, micafungin.

^a^Isolate DPLRG101 demonstrated a WT phenotype only to AFG and MFG and therefore data from ten isolates are presented for CAS and eleven isolates for AFG and MFG.

^b^Isolate DPLRG101 demonstrated a non-WT phenotype only to CAS and therefore data from four isolates are presented for CAS and three isolates for AFG and MFG.

### Macroscopic method

**Figure 2.**
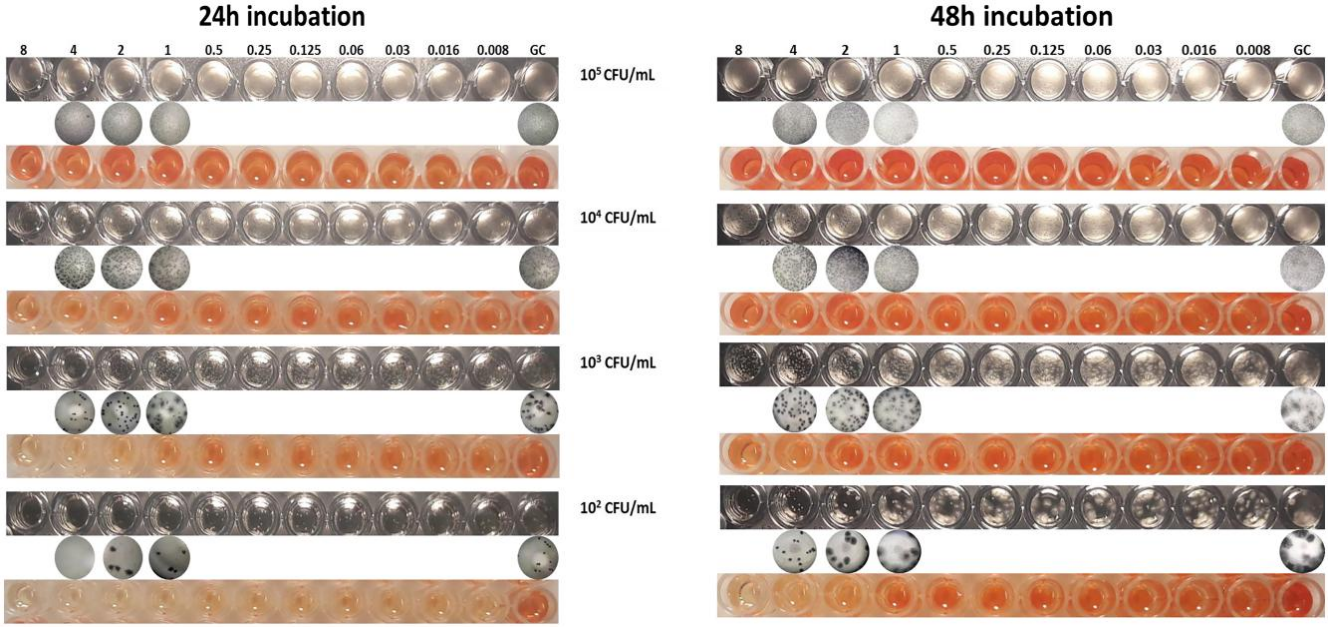
Macroscopic (first row), microscopic (second row) and XTT (third row) evaluation of growth of the atypical non-WT *A. fumigatus* isolate DPL32458^16^ exposed to 2-fold seral dilutions of anidulafungin (0.008–8 mg/L) using different inoculum sizes and time of incubation. This figure appears in colour in the online version of *JAC* and in black and white in the print version of *JAC*.

Similarly, the visual MECs were more clearly determined following 48 h of incubation. Macroscopic evaluation of growth using a 10^5^ cfu/mL inoculum resulted only in a slight decrease in turbidity for all drugs (Figure 1, first column). Using a 10^4^ cfu/mL inoculum, more distinct changes were observed, but aberrant mycelia as well as the mMEC endpoint were only visible to the naked eye with the 10^3^ and 10^2^ cfu/mL inocula. Notably, low EA (average 25%) between the microscopic and the visual MECs was found for the 10^5^ cfu/mL inoculum, while 4/10 WT strains were misclassified by both observers as non-WT to all three echinocandins, resulting in 71% overall CA, indicating the poor performance with the standard inoculum. On the contrary, the 10^2^ to 10^4^ cfu/mL inocula yielded 93%–100% EA for all echinocandins and all isolates could be correctly classified as WT/non-WT (100% CA) (Table 1). Of note, the non-WT isolates DPL55985 and DPL32458 demonstrated a unique pattern of growth with low (10^3^ and 10^2^ cfu/mL) inocula since small crippled rosettes were observed in both drug-containing and GC wells (Figure 2, second row). As the MEC is determined in comparison to GC growth, those two isolates should be considered non-WT considering that the growth pattern was the same in drug-containing and GC wells. The average (among the three drugs) interobserver agreement for MECs was 45%, 81%, 100% and 100% with the 10^5^, 10^4^, 10^3^ and 10^2^ cfu/mL inoculum, respectively.

### Spectrophotometric method

**Figure 3.**
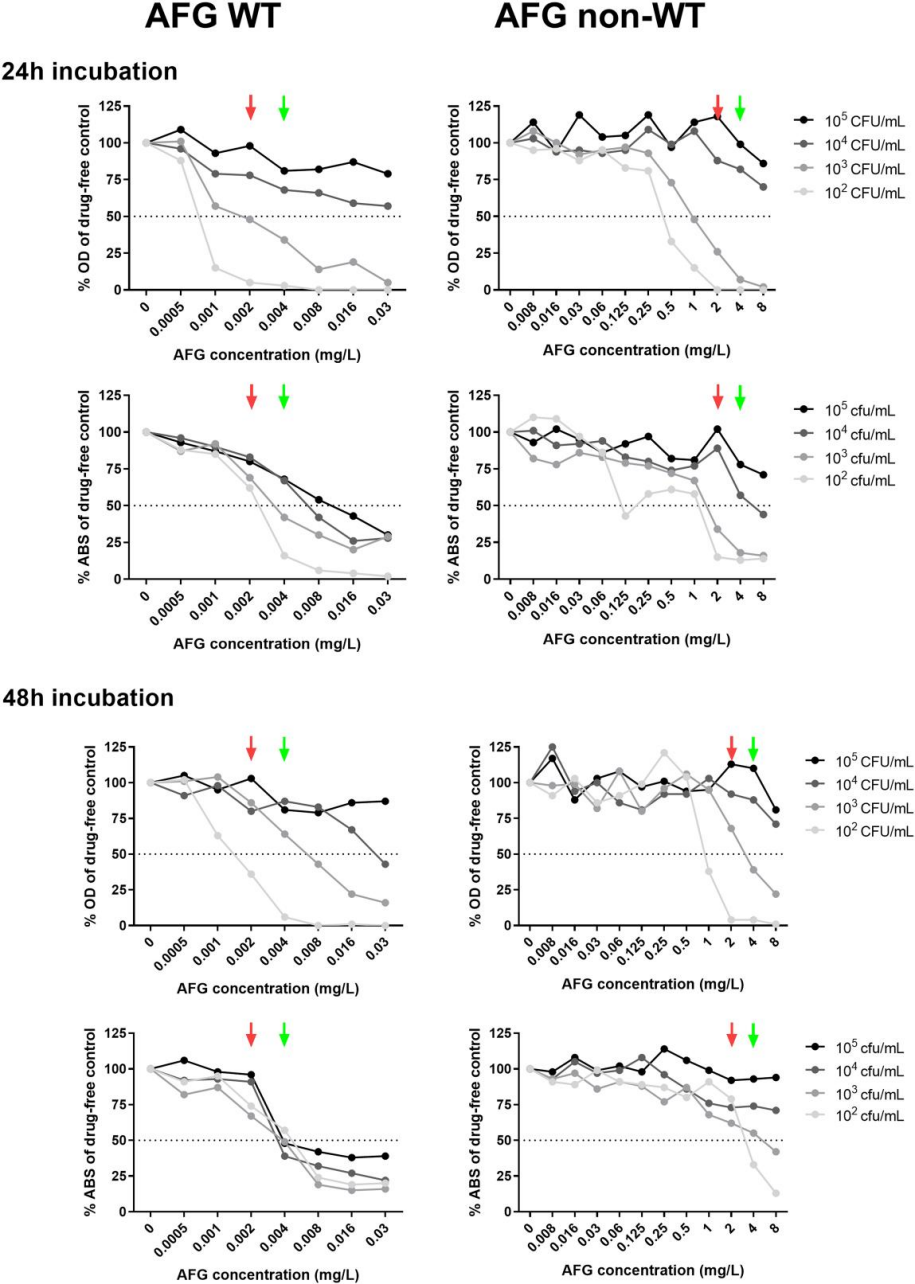
Spectrophotometric (OD at 405 nm) and colorimetric (ABS after XTT conversion by viable fungi measured at 450/630 nm, 2 h incubation with 400 mg/L XTT/6.25 μM menadione) concentration–effect curves of a WT *A. fumigatus* (left) and a non-WT *A. fumigatus* (right) exposed to anidulafungin (AFG) using different inoculum sizes and time of incubation. Green and red arrows represent microscopic MEC (mycelial rosettes) and mMEC (mycelial rosettes and healthy hyphae), respectively. The microscopic and macroscopic evaluations of growth of the same isolates are presented in Figure 1. Horizontal dotted lines corresponds to 50% growth. This figure appears in colour in the online version of *JAC* and in black and white in the print version of *JAC*.

Representative spectrophotometric curves using different inoculum sizes and time of incubation are illustrated in Figure 3.

After 24 h, the EA between the microscopic and spectrophotometric MECs and the CA in distinguishing WT from non-WT isolates using a 10^5^/10^4^ cfu/mL inoculum was ≤64% for all drugs, whereas the insufficient growth observed for a significant proportion of isolates using the 10^3^ and 10^2^ cfu/mL inocula precluded comparisons. After 48 h, the spectrophotometric readings did not produce a clear-cut endpoint for the highest (10^5^ and 10^4^ cfu/mL) inocula since <50% growth inhibition compared with the GC was found for most WT and non-WT isolates, making differentiation impossible. The EA between the microscopic and spectrophotometric MECs and CA using a 10^5^/10^4^ cfu/mL inoculum was ≤64% for all drugs, indicating that the microscopic changes caused by echinocandins are not detected by spectrophotometric measurements.

On the other hand, the EA between the microscopic and spectrophotometric methods for a 10^3^ cfu/mL inoculum following 48 h of incubation was 71% for anidulafungin, 93% for caspofungin and 57% for micafungin (*t*-test *P *= 0.56). The non-WT isolates had high spectrophotometric MECs (≥2 mg/L) for all three echinocandins (except micafungin and anidulafungin with DPLRG101 which was non-WT only to caspofungin). The CA in distinguishing WT from non-WT *A. fumigatus* isolates was 100% for caspofungin, and 86% for anidulafungin and micafungin since 2/11 WT strains were wrongly classified as non-WT with MEC > 0.03 mg/L (Table 2).

**Table 2.** Comparison of microscopic, spectrophotometric (based on 50% growth inhibition) and colorimetric (based on 50% inhibition of XTT conversion) MECs for WT and non-WT *A. fumigatus* isolates using the optimized test parameters (use of a 10^3^ cfu/mL inoculum and reading after 48 h of incubation)

		Median (range) MEC (mg/L)			% EA with the microscopic MEC (± 1 log_2_ dilution)		% CA with the microscopic MEC	
*A. fumigatus* isolates (*n*)	Drug	Microscopic	Spectrophotometric	Colorimetric	Spectrophotometric	Colorimetric	Spectrophotometric	Colorimetric
WT (10^a^)	AFG	0.004 (0.004–0.008)	0.008 (0.001 to >0.03)	0.008 (0.004–0.016)	64	100	82	100
	CAS	0.5 (0.5–1)	0.5 (0.25–1)	0.5 (0.5–1)	100	100	100	100
	MFG	0.008 (0.004–0.016)	0.016 (<0.0005 to >0.03)	0.008 (0.004–0.016)	55	100	82	100
non-WT (4^b^)	AFG	4 (4–4)	4 (2–4)	8 (8 to >8)	100	67	100	100
	CAS	8 (4–8)	8 (8 to >8)	8 (8 to >8)	75	75	100	100
	MFG	4 (2–4)	4 (4–8)	4 (4–4)	67	67	100	100
Total (14)	AFG				71	93	86	100
	CAS				93	93	100	100
	MFG				57	93	86	100

AFG, anidulafungin; CAS, caspofungin; MFG, micafungin.

^a^Isolate DPLRG101 demonstrated a WT phenotype only to AFG and MFG and therefore data from ten isolates are presented for CAS and eleven isolates for AFG and MFG.

^b^Isolate DPLRG101 demonstrated a non-WT phenotype only to CAS and therefore data from four isolates are presented for CAS and three isolates for AFG and MFG.

**Figure 4.**
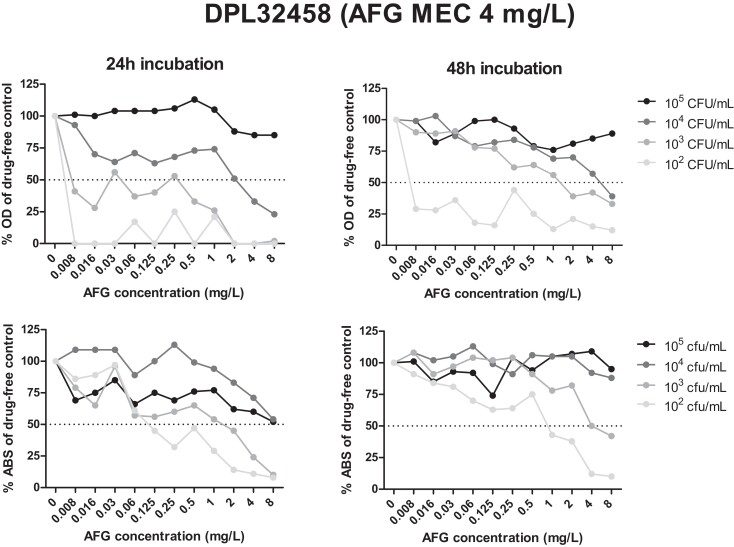
Spectrophotometric (OD at 405 nm) and colorimetric (ABS after XTT conversion by viable fungi measured at 450/630 nm, 2 h incubation with 400 mg/L XTT/6.25 μM menadione) concentration–effect curves of the non-WT *A. fumigatus* isolate DPL32458^16^ exposed to anidulafungin (AFG) for 24 h (left) and 48 h (right) using different inoculum sizes. Horizontal dotted lines corresponds to 50% growth.

Regarding the lowest (10^2^ cfu/mL) inoculum tested, the EA between the microscopic and spectrophotometric 48 h MECs was 57% for anidulafungin, 57% for caspofungin and 64% for micafungin (average 59%), while the overall CA was 86% as the non-WT isolates DPL55985 and DPL32458, which demonstrated crippled growth throughout the drug dilution range and in the GCs (Figure 2), were wrongly classified as WT to all three echinocandins (Figure 4).

### Colorimetric method

Representative colorimetric curves using different inoculum sizes and time of incubation are illustrated in Figure 3.

The EA between the microscopic and colorimetric MECs and the CA using a 10^5^/10^4^ cfu/mL inoculum was ≤36% and 79%–93% after 24 h, and ≤64% and 100% after 48 h for all drugs, respectively.

Regarding the 10^3^ cfu/mL inoculum, the non-WT isolates had higher colorimetric MECs (≥4 mg/L) than WT isolates for all three echinocandins except anidulafungin and micafungin against DPLRG101, which demonstrated a WT phenotype for the latter two drugs (Table 2). The EA between the microscopic and colorimetric methods and the CA was 93% and 100% for all three echinocandins after 48 h (*t*-test *P *= 0.37). Notably, the colorimetric 24 h MECs using a 10^3^ cfu/mL inoculum showed moderate EA (57%–71%) with the corresponding microscopic MECs for the 12/14 of isolates with sufficient growth attaining adequate metabolic activity (ABS > 0.8), and 100% overall CA.

Concerning the lowest (10^2^ cfu/mL) inoculum tested, the EA between the microscopic and colorimetric MECs and the CA was 64%–71% and 100% after 48 h for all drugs, respectively.

### Impact of Tween 20

No significant effect of Tween 20 concentration was found on microscopic/macroscopic and spectrophotometric/colorimetric MECs as all differences were within one 2-fold dilution.

## Discussion

Optimization of AFST methods is required to enable their implementation in the laboratory routine. A lower inoculum of 10^3^ cfu/mL facilitated visual MEC determination of echinocandins against *A. fumigatus* with the EUCAST methodology after 48 h of incubation, providing narrow MEC distributions, better agreement with the microscopic MECs and higher interobserver agreement and CA. The standard BMD growth-based technique could be automated relatively easily with the use of the optimized XTT assay, which could also be used for the detection of echinocandin resistance, even after 24 h of incubation if the metabolic activity of the GC is >0.8. No effect of Tween 20 concentration on MECs was found. The 24 h readings using high inoculum densities (10^5^/10^4^ cfu/mL) were challenging and MEC agreement was not improved with spectrophotometric/colorimetric readings, while a significant proportion of isolates did not grow sufficiently using a 10^3^/10^2^ cfu/mL inoculum.

A recent multicentre study demonstrated that the standard EUCAST protocol for MEC determination of *Aspergillus* spp. requires optimization. Using a high inoculum of 10^5^ cfu/mL, subtle differences of fungal growth between wells with different concentrations of echinocandins cannot be detected microscopically, yielding significant intercentre variation, even for experienced mycologists.^12^ Based on the aforementioned, we investigated the effect of incubation time and increasing inocula in an attempt to facilitate the EUCAST MEC determination for *A. fumigatus*. Some isolates, particularly the non-WT isolates, failed to produce sufficient growth after 24 h of incubation. In general, sufficient growth was evident for all isolates tested after incubation for 48 h. In fact, MECs tended to remain consistent (±1 2-fold dilution) for fast-growing isolates as the incubation period was extended from 24 to 48 h, as previously described.^17^ Echinocandin-induced morphological changes could not be identified microscopically and wide MEC ranges were recorded using the standard 10^5^ cfu/mL, in line with previous findings.^12,18^ On the other hand, obvious changes in microscopic MECs were noted and narrow MEC distributions were generated with lower inoculum sizes, with differences being more pronounced with 10^2^ and 10^3^ cfu/mL, enabling the MEC determination even for less experienced observers (interobserver agreement 82% for 10^4^ cfu/mL versus 100%/100% for 10^2^/10^3^ cfu/mL).

A 10^5^ cfu/mL inoculum results in heavy fungal growth of *Aspergillus* spp. after 48 h of incubation, impeding accurate macroscopic EUCAST MEC determination.^12^ Indeed, the EA between microscopic and visual MECs, as well as the interobserver agreement, were low (25% and 45%, respectively) and almost half of the WT isolates were wrongly classified as non-WT using the standard inoculum. On the contrary, the convenient visual MEC procedure could be suitable for evaluating the *in vitro* activity of echinocandins against *Aspergillus* spp. with a 10^3^ cfu/mL inoculum. In particular, both aberrant mycelia and an additional endpoint (mMEC defined microscopically by the presence of short, stubby and highly branched hyphal clusters together with healthy hyphae, and macroscopically by the presence of both pinpoint mycelial colonies and growth as in the GC with slight haziness inside the well as shown in red circled wells in Figure 1) could be easily detected by the naked eye, leading to excellent (100%) microscopic–macroscopic EA, CA and interobserver agreement for all drugs and isolates providing a practical way of determining MECs compared with the labour-intensive microscopic method. Notably, similar results were obtained for the 10^2^ cfu/mL; however, the use of such a low inoculum size (∼20 cfu/well) may result in low numbers of conidia inside the well if the inoculum is not prepared correctly. Detailed morphological changes induced by increasing drug concentrations allowing the determination of mMEC were not visible with a 10^4^ cfu/mL inoculum, while the interobserver agreement for MECs was moderate (81%). The mMECs can be used to quantify subMEC effects. The mMECs were closer to MECs for caspofungin than for anidulafungin and micafungin, indicating that caspofungin has a smaller subMEC effect than the other two echinocandins, as previously found in *in vitro* pharmacokinetic/pharmacodynamic studies.^19^ Moreover, previous studies have shown that a high concentration of Tween 20 (>0.1%) had a significant influence on the MIC determination of *Aspergillus* spp. against azoles and amphotericin B, but the magnitude of the effect is species-dependent as well as drug-dependent.^20^ Our data demonstrate that Tween 20 at final concentrations obtained during inoculum preparation did not affect echinocandin MEC values for *A. fumigatus*.

In order to increase objectivity and overcome the need for experienced laboratory personnel, the performance of spectrophotometric as well as colorimetric assays in EUCAST MEC determination was evaluated. Spectrophotometric (50% growth inhibition) and microscopic MECs showed a low level of EA (7%–14%), as well as CA (36%–43%), using a 10^5^ cfu/mL inoculum, as previously described.^12^ Nevertheless, when a lower inoculum size (10^3^ cfu/mL) was used, both the EA and the CA increased significantly (57%–93% and 86%–100%, respectively). On the other hand, higher (93%) EA was found between the microscopic method and the XTT assay (50% metabolic activity inhibition) using a 10^3^ cfu/mL inoculum, whereas the CA in distinguishing WT from non-WT isolates was 100%. Like the microscopic method, the colorimetric method provided narrow MEC distributions for all three echinocandins (1–3 2-fold dilutions), as opposed to the spectrophotometric MEC readings (2–9 2-fold dilutions). Furthermore, mMECs could not be defined spectrophotometrically (EA < 25%), demonstrating that morphological alterations induced by echinocandins cannot be detected in detail by the spectrophotometric assessment of fungal growth. In contrast, moderate (55%–80%) EA between the microscopic and colorimetric mMECs was recorded when the 75% metabolic activity inhibition was used as a cut-off. Importantly, the XTT assay could also be used for the detection of echinocandin non-WT isolates after 24 h of incubation (100% CA) provided that the GC had attained sufficient metabolic activity (ABS > 0.8). Nevertheless, this may not be feasible in the daily laboratory routine from a practical point of view and taking into account the risk for contamination since AFST is often conducted against various antifungals on the same panel and EUCAST recommends 48 h incubation of the microdilution plates for the MIC reading of azoles and amphotericin B.^11^

In conclusion, a 10^3^ cfu/mL inoculum after 48 h of incubation improved the microscopic and macroscopic MEC readings for the reference EUCAST BMD method E.Def 9.4, while the colorimetric assay could allow complete automation. However, a separate inoculation step will be required, diluting by 1/100 the standard inoculum of 10^5^ cfu/ml used for the other drugs. Since only 14 isolates have been tested, further studies with larger collections are required to verify findings. The study is limited to *A. fumigatus* since it remains the most prevalent species found in clinical settings, whereas acquired echinocandin resistance has not yet been reported in non-*A. fumigatus* spp.^5,6^ A multicentre collaborative study is needed to validate our results and assure reproducibility and standardization.
